# LC/MS Guided Isolation of Alkaloids from Lotus Leaves by pH-Zone-Refining Counter-Current Chromatography

**DOI:** 10.3390/molecules16032551

**Published:** 2011-03-18

**Authors:** Xia Xu, Cui-Rong Sun, Xiao-Jing Dai, Rui-Lin Hu, Yuan-Jiang Pan, Zhang-Fa Yang

**Affiliations:** 1 Department of Chemistry, Zhejiang University, Hangzhou 310027, China; E-Mails: daodaograce@gmail.com (X.X.); 20906164@zju.edu.cn (X.-J.D.); huruilin@zju.edu.cn (R.-L.H.); panyuanjiang@zju.edu.cn (Y.-J.P.); 2 Zhejiang Hisoar Pharmaceutical Co., LTD., No.100, Waisha Branch Rd., Jiaojiang District, Taizhou 318000, China; E-Mail: xxmuyang@126.com

**Keywords:** *Nelumbo nucifera* Gaertn., alkaloids, minor component separation, pH-zone-refining counter-current chromatography

## Abstract

The traditional methods used in natural product separation primarily target the major components and the minor components may thus be lost during the separation procedure. Consequently, it’s necessary to develop efficient methods for the preparative separation and purification of relatively minor bioactive components. In this paper, a LC/MS method was applied to guide the separation of crude extract of lotus (*Nelumbo nucifera* Gaertn.) leaves whereby a minor component was identified in the LC/MS analysis. Afterwards, an optimized pH-zone-refining CCC method was performed to isolate this product, identified as *N*-demethylarmepavine. The separation procedure was carried out with a biphasic solvent system composed of hexane-ethyl acetate-methyl alcohol-water (1:6:1:6, v/v) with triethylamine (10 mM) added to the upper organic phase as a retainer and hydrochloric acid (5 mM) to the aqueous mobile phase eluent. Two structurally similar compounds – nuciferine and roemerine – were also obtained from the crude lotus leaves extract. In total 500 mg of crude extract furnished 7.4 mg of *N*-demethylarmepavine, 45.3 mg of nuciferine and 26.6 mg of roemerine with purities of 90%, 92% and 96%, respectively. Their structures were further identified by HPLC/ESI-MS^n^, FTICR/MS and the comparison with reference compounds.

## 1. Introduction

Medicinal plants are viewed as important sources of new drugs since they contain numerous compounds with significant pharmacological potential, many of which may serve as lead compounds in the development of new drugs. Consequently, it is important to be able to separate and identify these bioactive compounds, but traditional separation methods have several drawbacks: (1) they primarily target major components; (2) minor components might be lost during the separation procedure; (3) bioactive components may be deactivated. Hence development of effective methods to discover bioactive compounds in natural sources is deemed necessary. Liquid chromatography tandem mass spectrometry (LC/MS) has been widely used to analyze complex mixtures, such as biological samples [1,2,3]. This protocol is very effective at identifying a large number of constituents in a sample, especially when looking for minor components.

Counter-current chromatography (CCC) is a solid support-free liquid-liquid partition chromatography technique [4] that eliminates the complications resulting from the use of a solid support matrix, such as irreversible adsorptive sample loss and deactivation, tailing of solute peaks, and contamination. CCC has been widely used as a preparative separation method in natural products chemistry [5,6,7]. Developed by Ito in the 1990s [8,9], pH-zone-refining CCC enables separation of organic acids and bases into a succession of highly concentrated rectangular peaks that elute according to their corresponding pK_a_ values and hydrophobicities. So far, this method has been applied successfully to the analysis and separation of various natural and synthetic products, especially alkaloids and organic acids [10,11,12,13]. It has many important advantages compared with traditional CCC, including an over 10-fold increase in sample loading capacity, high concentration of fractions, concentration of minor impurities, *etc.*

*Nelumbo nucifera* Gaertn. of the family *Nelumbonaceae*, is a perennial, rhizomatous, aquatic plant widely spread throughout southeastern China [14]. Lotus leaves have been used as a foodstuff and in Traditional Chinese Medicine to clear heat, resolve summer heat and stop bleeding [15]. According to previous studies, the major components of lotus leaves are alkaloids such as nuciferine, roemerine, and *N*-demethylarmepavine [16]. These alkaloid compounds have been reported to show remarkable biological activities, including antioxidant [17], antimicrobial [18], anti-HIV [15], anti-hyperlipidemic [19], anti-obesity [20,21], antiplatelet [22] and hypotensive [23] properties. 

The preparative separation and purification of alkaloids from lotus leaves by conventional methods is tedious and complicated. Consequently, in order to further study the intrinsic activity of these alkaloids and their pharmacological mechanism(s), an efficient method for the preparative separation and purification of these active compounds is necessary. The main goal of this paper was to establish an effective method to separate the relatively minor bioactive components. First, the LC/MS technique was used to screen the natural product. Afterwards, pH-zone refining CCC was successfully applied to the preparative isolation and purification of target alkaloids from an ethanol lotus leaves extract. Finally, the structures were further reconfirmed by HPLC/ESI-MS^n^ and high resolution mass spectrometry using reference compounds.

## 2. Results and Discussion

### 2.1. LC/MS/MS analysis

**Figure 1:**
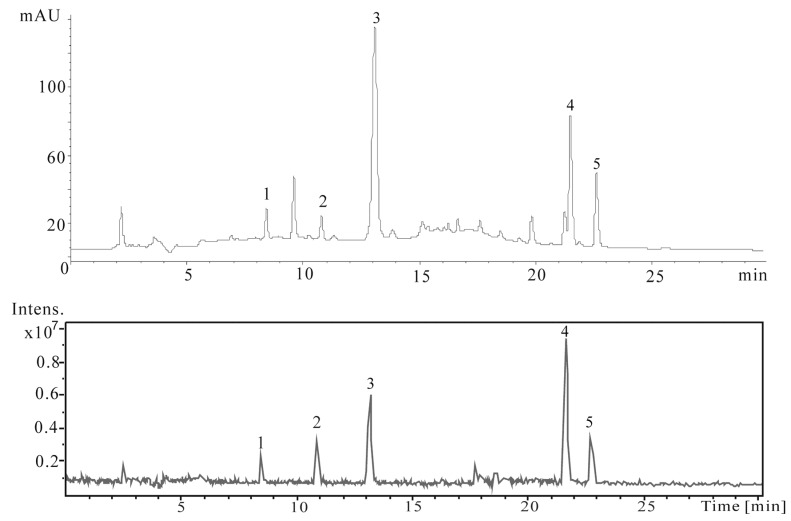
HPLC chromatogram obtained by UV detection at 280 nm and TIC chromatogram of the crude extract from lotus leaves in the positive ESI-MS mode.

The crude extract obtained from lotus leaves was first analyzed by HPLC/ESI-MS^n^. The HPLC/UV detection and total ion chromatogram (TIC) are shown in Figure 1. As shown in the figure, the crude extract used in present study is highly complex, containing a number of compounds with a wide range of hydrophobicities. The LC/MS analysis provides the molecular weight information for the components of the extract. MS*^2^* dissociations give further structural information on the target compounds, as listed in Table 1, complementing the high-resolution FTICR-MS data listed in Table 2. 

**Figure 2:**
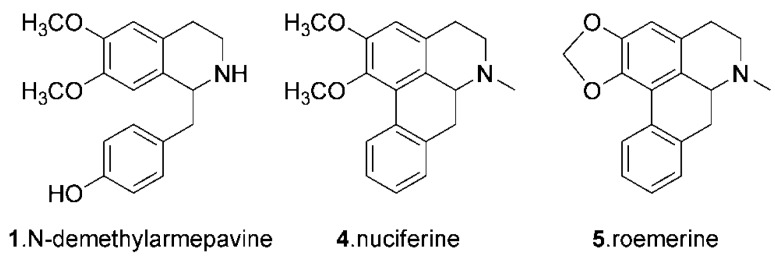
Chemical structures of target alkaloids in this study.

The full-scan mass spectrum of compound **1** showed a protonated ion at *m/z* 300. In the MS/MS spectrum of the [M+H] ^+^ ion of this compound, three fragment ions at *m/z* 283, 189 and 107 were observed. The first two correspond to losses of 17 and 111 Da, respectively, while the ion at *m/z* 107 corresponds to a benzyl moiety. All these fragments were also observed in the mass spectrum of protonated norcoclaurine, which was previously investigated by Schmidt [24], suggesting that the structure of compound **1** was similar to that of norcoclaurine. Meanwhile, the fragmentation behaviors of protonated compounds **3, 4** and **5** were the same as those previously published for *O*-nornuciferine, nuciferine and roemerine (Figure 2) [25]. The MS^2^ ions produced by compound **2** were similar with those of compound **1**. Compared with a previous study [13], compound **2** might be armepavine. Based on the comparison of the retention times and the fragmentation behaviors observed in the MS^n^ experiments, compound **1** could be identified as *N*-demethylarmepavine (Figure 2). 

As mentioned above, the relatively minor compound **1** (about 2% in the HPLC analysis) has valuable bioactivities [22,23]. In addition, its pharmacological mechanism is still not clear and requires further investigation. Thus, preparative isolation of compound **1** from medicinal plants to obtain a reference standard and material for further study is important. During the traditional separation procedure, most of the minor components are actually lost due to the adsorptive process of the solid matrix, whereas pH-zone refining CCC could avoid the irreversible adsorptive sample loss because of the absence of the solid support. Since additionally there were no reports of the separation of this compound using CCC, we therefore tried to separate compound **1** from the extract of lotus leaves by pH-zone refining CCC. 

### 2.2. pH-zone refining CCC separation

**Figure 3:**
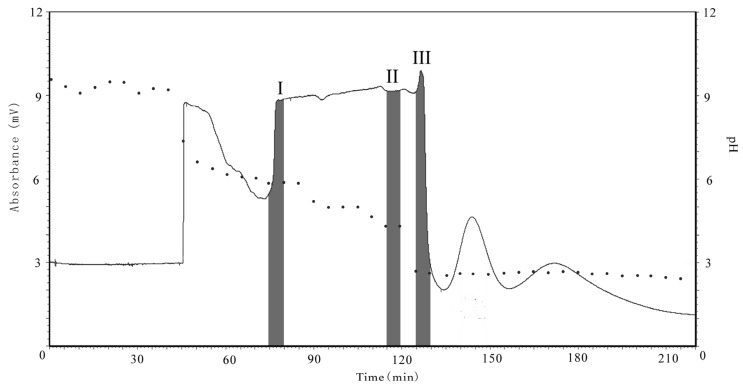
pH-zone-refining counter-current chromatogram (solid line) and pH value (dashed line) for each CCC fraction. Experimental conditions: solvent system: hexane-ethyl acetate-methyl alcohol-water (1:6:1:6, *v/v*), including 10 mM TEA in the upper organic stationary phase and 5 mM HCl in the lower phase; retention of stationary phase: 34%; flow-rate: 2.0 mL/min; UV detection: 254 nm; revolution speed: 600 rpm.

**Figure 4:**
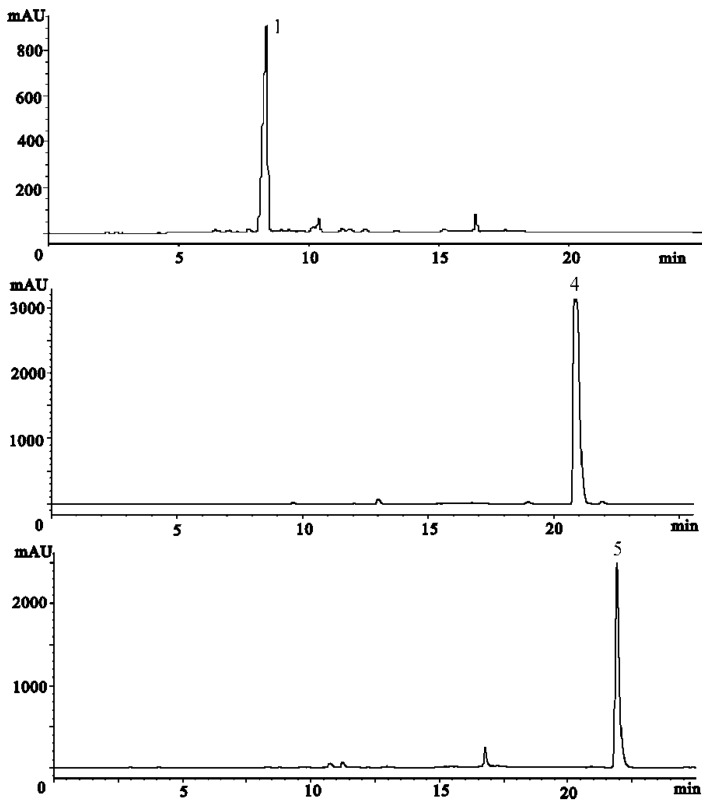
HPLC analysis of CCC fractions I-III.

The second part of this study focuses on the separation of the target compounds by pH-zone refining CCC. Optimization of the biphasic solvent system is very important for a successful CCC separation. According to the existing applications of pH-zone refining CCC to alkaloids, a suitable two-phase solvent system which should provide ideal partition coefficient (K) values in both acidic (K_acid_<<1) and basic (K_base_>>1) conditions as well as good solubility of samples in the solvent systems was deemed necessary. We first evaluated a binary two-phase solvent system composed of MtBE-CH_3_CN-water (2:2:3, *v/v*), which has been widely used in pH-zone refining mode [26]. Although this system provided good solubility, the K values were not good enough for our separation. Afterwards, the solvent system was optimized by selecting hexane-ethyl acetate-methyl alcohol-water (1:6:1:6, *v/v*). The K value was suitable for separation and the crude extract also dissolved well in this solvent system. Figure 3 shows a typical preparative pH-zone-refining CCC separation of 500 mg of crude extract, using the optimized hexane-ethyl acetate-methyl alcohol-water solvent system. Each CCC fraction was analyzed by HPLC. The corresponding chromatograms are shown in Figure 4. As a result, 7.4 mg of material corresponding to *N*-demethylarmepavine (**1**) was successfully separated, together with larger amounts of the two compounds **4**-**5** (45.3 mg of nuciferine and 26.6 mg of roemerine, respectively). Compounds **1**, **4** and **5** were thus obtained from zones I, II, and III, respectively, with purities of 90%, 92% and 96% in a single separation step.

The pH-zone refining CCC is usually used as a preparative protocol to separate the major components in extracts. Zheng *et al.* isolated three major components present in large amounts in lotus leave extract, which proved that the major components in lotus leaves can be well separated by pH-zone refining CCC [20]. Compared with Zheng’s work, the plant extract here was more complex and the separation time was shorter. In addition, a minor component was separated. Searching the reports of the application of pH-zone refining CCC, there are relatively few reports of separation of the minor components. Besides, two structurally similar compounds such as nuciferine and roemerine were separated successfully, indicating that pH-zone refining CCC has great potential in the purification of the relatively low abundance alkaloids and structurally similar compounds from medicinal plants.

## 3. Experimental 

### 3.1. Reagents and materials

Hexane, ethyl acetate, methanol, hydrochloric acid and triethylamine (TEA) for CCC were of analytical grade and purchased from Huadong Chemicals, Hangzhou, China. Reverse osmosis Milli-Q water (18 MΩ) (Millipore, Bedford, MA, USA) was used for all solutions and dilutions. Acetonitrile for HPLC analysis was of chromatographic grade and purchased from Merck, Darmstadt, Germany. Lotus leaves were purchased from a local drugstore in Hangzhou. The reference substances (see Figure 3) were bought from the National Institute for the Control of Pharmaceutical and Biological Products, Ministry of Health, Beijing, China.

### 3.2. Apparatus

#### 3.2.1. Counter-current chromatography

The separations were performed on a semi-preparative apparatus (Ito scheme IV) with one 140 mL coil and a counterweight. This instrument was manufactured at the Zhejiang University machine shop (Hangzhou, China). The multilayer coil was prepared by winding a 26.4 m × 2.6 mm i.d. PTFE tube. The *β*-value varied from 0.33 at the internal terminal to 0.60 at the external terminal (*β* = *r/R* where *r* is the distance from the coil to the holder shaft, and *R* is the revolution radius or the distance between the holder axis and central axis of the centrifuge). The revolution speed can be regulated with a speed controller in the range of 0–1,000 rpm able to produce 100 g gravitational field at most. Sample injection was accomplished by an injection valve with a 10 mL sample loop. A Model 2W-2B constant-flow pump (Beijing Xingda Equipment, Beijing, China) was used to fill the CCC apparatus with the stationary phase and elute the mobile phase. The effluent was continuously monitored by an HD-9704 UV spectrometer (Jingke Equipment, Shanghai, China) operating at 254 nm. Eluent was collected by a BSZ-100 fraction collector and a N2000 data analysis system (Institute of Automation Engineering, Zhejiang University, Hangzhou, China) was used to record the CCC chromatograms. A PHS-3B pH Meter (Shanghai Precision & Scientific instrument, Shanghai, China) was used for pH measurements.

#### 3.2.2. HPLC 

An Agilent 1100 analytical HPLC system with a G1312 Binary pump, a G1314A variable-wavelength detector (VWD), a model 7725 injector fitted with a 20 μL sample loop, along with an Agilent ChemStation data system, was used. A Hypersil reverse phase C18 column, (250 mm × 4.6 mm i.d., 5 μm, Yilite) was used for separation; the column was maintained at room temperature. HPLC analysis of the crude extract and CCC fractions was carried out in the gradient mode with acetonitrile (solvent A) and 0.1% TEA in water (solvent B). The percentage of solvent A was changed linearly as follows: 0 min 20% A; 5 min 30% A; 10 min 30% A; 13 min 60% A; 20 min 80% A. The flow rate was 0.8 mL/min and the chromatograms were monitored at 280 nm.

#### 3.2.3. Mass spectrometry

HPLC/ESI-MS^n^ analysis were performed using the Agilent HPLC system described above combined with a Bruker Esquire 3000^plus^ ion trap mass spectrometer (Bruker-Franzen Analytik GmbH, Bremen, Germany) equipped with an electrospray ionization source (ESI). Instrument control and data acquisition were performed using Esquire 5.0 software. The ion source temperature was 250 °C, and the needle voltage was always set at 4.0 kV. Nitrogen was used as the drying and nebulizer gases at a flow rate of 10 L/min and a backpressure of 30 psi. 

The offline FTICR-MS experiments were performed using an Apex III Fourier transform ion cyclotron resonance mass spectrometer with 7.0 T actively shielded superconducting magnet (Bruker Daltonics, Billerica, MA, USA) combined with an Apollo electrospray ionization source operated in the positive ion mode. The solutions were infused at a rate of 3.0 μL/min using a Cole-Parmer syringe pump. Each spectrum was an average of eight transients, each composed of 512 K points, acquired using a workstation operating XMASS version 6.1.1.

### 3.3. Preparation of crude extract

The ethanolic lotus leaves extract was dissolved with 1% HCl (400 mL). After removing the residue, the pH value of the acidic aqueous solution was adjusted to 10 with sodium hydroxide. Then the residue as well as the aqueous solution was extracted three times with chloroform. The extract was combined and evaporated to dryness by rotary vaporization under reduced pressure at 45 °C. 600 mg crude extract was obtained and stored in a refrigerator (4 °C) for the subsequent pH-zone-refining CCC separation.

### 3.4. Preparation of the two-phase solvent system and sample solution

The two-phase solvent system used for CCC separation was thoroughly equilibrated in a separatory funnel at room temperature and the two phases were separated. TEA was added to the upper phase at 10 mM as a retainer and HCl was added to the lower phase at 5 mM as an eluter. The sample solution was prepared by dissolving the crude extract in 5 mL of the upper phase.

### 3.5. Separation procedure

For each separation, the CCC column was first entirely filled with the upper organic phase. Then the crude extract, dissolved in the upper phase was injected through the sample port and the lower mobile phase was pumped through the column at a flow rate of 2.0 mL/min in the head to tail direction (reversed phase), while the column was rotated at 600 rpm. The effluent was monitored continuously at 254 nm and automatically collected in test tube per 5 min using a BSZ-100 fraction collector. After the separation was completed, retention of the stationary phase was measured by collecting the column contents into a graduated cylinder after forcing them out of the column with pressurized nitrogen gas. Peak fractions were analyzed by HPLC. The retention of stationary phase was 34%.

## 4. Conclusions

In this work, HPLC/ESI-MS^n^ was applied to analyze crude lotus leaves extract and a minor component with the molecular weight of 299 was found. Afterwards, the CCC separation conditions for the target minor alkaloid were optimized. Finally, a pH-zone-refining CCC method was successfully applied to isolate the target, identified as *N*-demethylarmepavine. Meanwhile, two structurally similar compounds – nuciferine and roemerine – were also separated from the crude lotus leaves extract. The results demonstrated that pH-zone-refining CCC is an effective technique not only for the separation of major components but also for the separation of minor components.

## Figures and Tables

Column: Hypersil reverse phase C_18_ column, (250 mm × 4.6 mm i.d., 5 μm, Yilite); mobile phase: acetonitrile (solvent A)-0.1% TEA in water (solvent B) at the following gradient: 0 min 20% A; 5 min 30% A; 10 min 30% A; 13 min 60% A; 20 min 80% A.; UV detection: 280 nm; flow rate: 0.8 mL/min.

**Table 1:** Main MS^2^ fragments of compounds **1**–**5**.

Compounds	[M+H]^+^	Product ions *m/z* (Relative Abundance, %)
**1**	300	283(100), 189(8), 107(7)
**2**	314	283(100), 189(20),107(13)
**3**	282	251(100), 219(62)
**4**	296	265(100)
**5**	280	249(100)

**Table 2:** Accurate masses and assigned elemental compositions of **1**, **4** and **5**.

Compounds	Composition	Measurement (*m/z*)	Theoretical (*m/z*)	Error (ppm)
**1**	C_18_H_21_NO_3_H^+^	300.1596	300.1594	+0.7
**4**	C_19_H_21_NO_2_H^+^	296.1637	296.1645	−2.7
**5**	C_18_H_17_NO_2_H^+^	280.1332	280.1332	0
